# Antifungal activity of essential oil of *Amyrisbalsamifera *against *Cryptococcusneoformans*

**DOI:** 10.1186/1753-6561-8-S4-P32

**Published:** 2014-10-01

**Authors:** Fernando Abrão, Lorena Peixoto, Marcelo Ramada, Mariana Oliveira, Ana Flávia Mendonça, Carolina Treméa, Lucia Souza, Maria Silva

**Affiliations:** 1 Instituto de Patologia Tropical e Saúde Pública (IPTSP), Universidade Federal de Goiás, 74605-050, Goiânia-GO, Brazil; 2 EMBRAPA Arroz e Feijão, 75375-000, Santo Antônio de Goiás-GO, Brazil; 3 EMBRAPA Recursos Genéticos e Biotecnologia - Parque Estação Biológica, 70770-910, Brasília-DF, Brazil

## Background

*Cryptococcus neoformans *can cause infection in immunocompromissed individuals, especially in patients with acquired immunodeficiency syndrome, in which meningoencephalitis is the main clinical manifestation [1]. There are few available antifungals for cryptococcosis treatment and all of them present high toxicity, besides the reports of resistance. In this context, the natural products from plants are an important source in the search for new antifungal compounds. In this study, it was evaluated the antifungal activity of the *Amyrisbalsamifera *essential oil (EO) against *C. neoformans*. The determination was made by minimum inhibitory concentration (MIC) and minimum fungicidal concentration (MFC) using the microdilution broth method.

## Methods

Broth microdilution protocols based on the CLSI reference document M27-A3 [2], were used to determine MIC values for 15 isolates of *C. neoformans*. Briefly, twofold serial dilutions, in Roswell Park Memorial Institute (RPMI 1640) broth, with final test concentrations ranging from 2 to 1024 µg/mL for *A. balsamifera *was tested, and the inocula was prepared in the same broth with 10^3 ^yeasts/mL. Results were read after 72 h, and MICs were defined as the lowest test concentrations causing complete growth inhibition. Quality control determinations of the MIC values of fluconazole were performed by testing *Candida parapsilosis *ATCC 22019 and the results obtained were within the recommended limits. To determine minimum fungicidal concentration (MFC) [3] values, after reading the corresponding MIC values, 10 µl samples from all optically clear tubes (complete growth inhibition) plus the last tube showing growth were subcultured on Sabouraud Dextrose Aagar Petri dishes. The dishes were incubated at 35°C for for 3 days, until growth was clearly visible in the control samples, and MFC values were determined as the lowest concentration EO which there was no visible growth.

## Results and conclusions

*A. balsamifera *EO exhibited wide-spectrum antifungal activity. Evaluation of MIC and MFC values showed that the EO was active against all the tested strains. MIC values ranged from 128 to 256 µg/mL against *Cryptococcus*, and MFC values were between 128 to 512 µg/mL. Acording to Scorzoni [4] compounds with MICs ≤ 256 µg/mL are considered relevant in the investigation of substances for therapeutic purposes, so in our study we can conclude that the EO showed antifungal activity against yeasts of the complex *C. neoformans*.
